# Microencapsulation of *Idesia polycarpa* Oil: Physicochemical Properties via Spray Drying vs. Freeze Drying

**DOI:** 10.3390/ijms27031363

**Published:** 2026-01-29

**Authors:** Yunhe Chang, Haocheng Yang, Bo Zeng, Mingfa Song, Juncai Hou, Lizhi Ma, Hongxia Feng, Yan Zhang

**Affiliations:** 1 School of Food Science and Engineering, Guiyang University, Guiyang 550005, China; 2 Engineering Technology Research Center for Processing and Comprehensive Utilization of *Idesia polycarpa* of National Forestry and Grassland Administration, Guiyang 550005, China; 3 Guizhou Collaborative Innovation Center for Fruit Processing, Storage and Safety Control, Guiyang 550005, China; 4 College of Horticulture and Landscape Architecture, Northeast Agricultural University, Harbin 150030, China

**Keywords:** *Idesia polycarpa* oil, lipid stabilization, volatile retention, shell structure, molecular diffusion

## Abstract

This study systematically compared spray drying (SD) and freeze drying (FD) for microencapsulating *Idesia polycarpa* oil using a soy protein isolate/maltodextrin (SPI/MD) wall system. SD produced predominantly spherical and compact microcapsules with higher solubility (51.33%), encapsulation efficiency (81.9%), and superior oxidative stability (oxidation induction period, 6.05 h), together with improved thermal resistance, indicating its suitability for applications requiring enhanced stability and aroma retention. In contrast, FD yielded irregular and porous microcapsules with significantly higher emulsifying activity (29.12 m^2^ g^−1^, *p* < 0.05) but lower solubility and encapsulation efficiency. Integrated physicochemical characterization-including scanning electron microscopy (SEM), Fourier transform infrared spectroscopy (FTIR), particle size and polydispersity index (PDI), ζ-potential, differential scanning calorimetry (DSC), oxidative stability index (OSI) measurements, and volatile profiling via odor activity value (OAV) analysis—revealed clear process-dependent structure–function relationships. The denser SPI/MD matrix formed during SD restricted lipid molecular mobility and oxygen diffusion, thereby suppressing lipid oxidation and promoting the retention of key lipid-derived odorants. Conversely, the porous structure generated by FD facilitated interfacial functionality but increased molecular diffusion pathways. Overall, this work demonstrates that SPI/MD-based microencapsulation functions as a molecular stabilization platform for highly unsaturated plant oils and provides mechanistic guidance for selecting drying strategies to tailor *Idesia polycarpa* oil microcapsules for specific food applications.

## 1. Introduction

*Idesia polycarpa* Maxim. (*I. polycarpa*) is a deciduous tree of the Salicaceae family and is widely distributed in Asia, including China, Korea, and Japan, with primary cultivation in southwestern China [1]. This species is extensively cultivated, produces abundant fruit, and exhibits a high oil content ranging from 21.27% to 39.47% in dried fruits [2]. Studies have identified that the oil contains unsaturated fatty acids, including linoleic acid, palmitic acid, and oleic acid, with linoleic acid comprising between 52.18% and 78.86% of the total fatty acids [3]. Furthermore, the refined oil contains bioactive compounds including vitamin E, squalene, and sterols [4]. Prior studies report lipid-modulating, antioxidant and antibacterial activities associated with these constituents [5,6], highlighting the oil’s nutritional and functional potential. However, regarding the unsaturated fatty acids of *I. polycarpa* oil, they are susceptible to degradation by environmental factors such as oxygen, light exposure, and heat, leading to a consequent reduction in nutritional value and safety. Therefore, processing strategies that preserve nutritional quality while improving stability are needed, such as protein–polysaccharide-based microencapsulation.

Microencapsulation has been increasingly investigated and applied to improve the stability and functionality of edible oils. By entrapping the oil within protein–polysaccharide wall matrices, oxygen diffusion and pro-oxidant contact are reduced, thereby enhancing oxidative stability, extending shelf life, and better preserving lipid-associated bioactives [7,8]. Representative studies show that walnut oil encapsulated with soy protein isolate (SPI) and oxidized konjac glucomannan (OKGM) exhibits improved oxidative stability and more favorable in vitro digestion behavior [9], and that sesame oil similarly benefits from microencapsulation in terms of quality retention [10]. Against this backdrop, comparing drying routes for microcapsule formation is pertinent, since process choice (e.g., spray drying vs. freeze drying) governs shell structure, surface oil exposure, and, ultimately, stability and functionality.

In drying-based microencapsulation, spray drying and freeze drying are the two most widely used and effective routes. Spray drying is broadly adopted in the food industry for its scalability and process efficiency [11,12]. It converts emulsions into microcapsules via atomization and rapid solvent removal, typically yielding compact, near-spherical microcapsules and accommodating a wide range of protein–polysaccharide wall matrices [13]. Representative studies on edible plant oils show that spray-dried microcapsules can enhance oxidative stability and quality retention—for example, in walnut and grape seed oils prepared with protein/polysaccharide walls [14,15].

Freeze drying removes water by sublimation under low temperature and vacuum, which helps preserve thermolabile constituents and typically generates porous, sponge-like matrices with large specific surface areas. Compared with spray drying, freeze-dried microcapsules often show higher redispersibility/interfacial activity but may exhibit greater surface-oil exposure due to the open pore network created by ice-crystal templating; these structural traits can impact oxidative stability and aroma retention [16,17]. In edible-oil systems where both routes were compared, spray-dried microcapsules frequently achieved higher encapsulation efficiency and lower surface oil, while freeze-dried counterparts emphasized milder thermal histories and porous structures [18,19]. Under protein–polysaccharide walls relevant to this work (e.g., SPI/MD), spray drying of plant oils has been shown to yield compact, near-spherical microcapsules with improved oxidative stability, whereas freeze drying preserves heat-labile components but can alter interfacial properties [14,20]. Despite the broad use of both routes, process-dependent differences in structure and functionality under the same wall formulation remain insufficiently resolved.

In addition, the interactions among SPI, MD, and the unsaturated lipids of *I. polycarpa* oil involve several molecular stabilization mechanisms. SPI can adsorb at the oil–water interface through hydrophobic interactions and hydrogen bonding, whereas maltodextrin forms a dense carbohydrate matrix that restricts molecular mobility and oxygen accessibility. These molecular-level interactions are further associated with the thermal transitions (DSC), chemical environments (FTIR), and oxidation behavior (OSI) observed in this study. By linking physicochemical results with these molecular phenomena, the present work provides a mechanistic interpretation of lipid stabilization in biopolymer-based microcapsules. Therefore, rather than serving solely as a comparison of spray drying and freeze drying processes, this study positions the SPI/MD microencapsulation system as a molecular stabilization platform for highly unsaturated lipids. By integrating physicochemical characterization with mechanistic interpretation, the work aligns with the scope of molecular material sciences and aims to elucidate how biopolymer–lipid interactions govern molecular mobility, oxidation pathways, and volatile retention. Overall, this work is framed within the scope of molecular materials science by elucidating how biopolymer matrices stabilize unsaturated lipids through molecular-level interactions, rather than merely comparing drying processes from a food technology perspective.

## 2. Results

### 2.1. Encapsulation Efficiency

The EE of *I. polycarpa* oil microcapsules produced by spray drying reached 81.9 ± 0.18%, which was significantly higher than that obtained by freeze drying (66.21 ± 0.21%) (*p* < 0.05). This result indicates that spray drying provides superior core material retention compared with freeze drying.

### 2.2. Physicochemical Properties of I. polycarpa Oil Microcapsules

Table 1 summarizes the physicochemical properties of *I. polycarpa* oil microcapsules prepared by spray drying and freeze drying, including solubility, moisture content, particle size, PDI, ζ-potential, emulsifying activity, and emulsion stability.

Spray-dried samples showed higher solubility (51.33 ± 0.08%) than freeze-dried ones (47.83 ± 0.19%) (*p* < 0.05). Moisture content was slightly lower for freeze-dried microcapsules (1.58 ± 0.09%) than for spray-dried microcapsules (1.76 ± 0.05%).

Dynamic light scattering (DLS) revealed unimodal particle size distributions for both methods. Freeze-dried microcapsules showed D10 = 538.80 ± 45.27 nm, D50 = 750.72 ± 93.55 nm, and D90 = 1087.35 ± 234.16 nm, with a PDI of 0.353 ± 0.22 (n = 3). Spray-dried samples exhibited larger hydrodynamic sizes (D10 = 1583.58 ± 352.89 nm, D50 = 1998.76 ± 515.77 nm, D90 = 2542.67 ± 732.55 nm) and a comparable PDI (0.385 ± 0.23) (n = 3).

The ζ-potential of spray-dried microcapsules was −40.50 ± 0.70 mV, compared with −36.76 ± 1.20 mV for freeze-dried microcapsules. Emulsifying activity (EAI) was higher after freeze drying (29.12 ± 0.53 m^2^ g^−1^) than after spray drying (24.15 ± 0.20 m^2^ g^−1^), whereas emulsion stability was higher for spray-dried microcapsules (95.06 ± 3.18% vs. 88.96 ± 4.78%).

### 2.3. Morphology of I. polycarpa Oil Microcapsules

**Figure 1:**
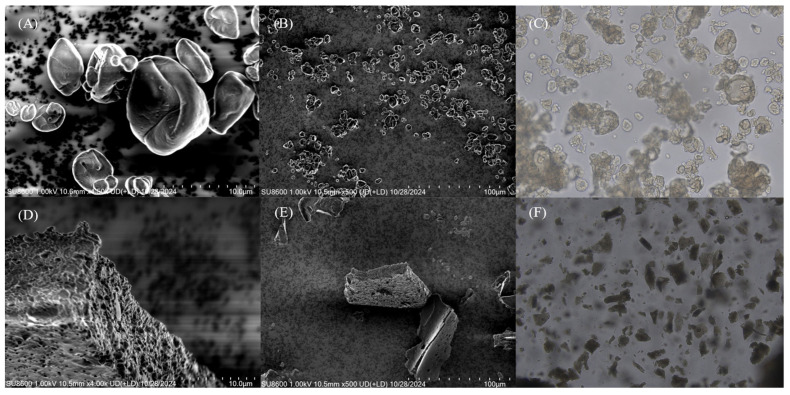
Microstructure of *I. polycarpa* oil microcapsules prepared by spray drying and freeze drying, observed by scanning electron microscopy (SEM) and optical microscopy. (**A**,**B**) SEM images of spray-dried microcapsules at low and high magnification, showing predominantly spherical particles with compact and smooth surfaces. (**C**) Optical microscopy image of spray-dried microcapsules (×400), confirming spherical morphology and uniform dispersion. (**D**,**E**) SEM images of freeze-dried microcapsules, revealing irregular, block-like structures with porous surfaces generated by ice-crystal sublimation. (**F**) Optical microscopy image of freeze-dried microcapsules (×400), showing fragmented and irregular particles corresponding to the porous morphology observed in SEM.

The microscopic morphology of *I. polycarpa* oil microcapsules prepared by different drying methods is presented in Figure 1. Spray-dried microcapsules (Figure 1A–C) exhibited predominantly spherical structures with smooth and continuous surfaces, and only slight shrinkage was observed. No cracks or fractures were detected, indicating the formation of an intact wall matrix. In contrast, freeze-dried microcapsules (Figure 1D–F) showed irregular, block-like structures with rough surfaces and pronounced porosity. The porous morphology is attributed to the sublimation of ice crystals during freeze drying, which produced voids within the wall matrix. Despite surface irregularities, the overall structural integrity of the freeze-dried samples remained intact without collapse.

### 2.4. Thermal Stability of I. polycarpa Oil Microcapsules

**Figure 2:**
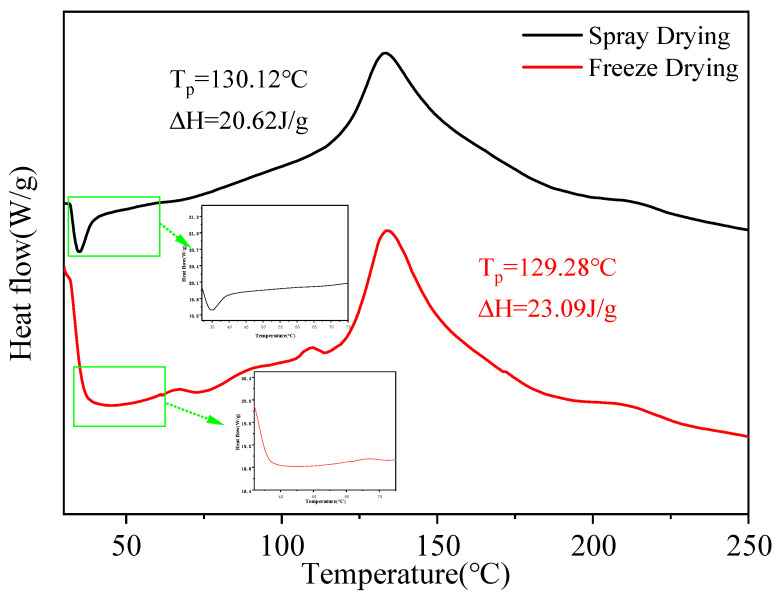
Differential Scanning Calorimetry (DSC) thermograms of spray-dried and freeze-dried *I. polycarpa* oil microcapsules.

The DSC thermograms of microcapsules prepared by freeze drying and spray drying are shown in Figure 2. Freeze-dried samples displayed multiple endothermic transitions. The minor peak at 67 °C corresponds to the melting of monoglycerides [21]. A distinct transition at 110 °C is associated with the denaturation of soybean protein isolate (SPI) [22], while the broad endothermic peak near 134 °C is attributed to structural changes in maltodextrin and the release of bound water [23]. The total enthalpy change was 23.09 J/g.

In contrast, spray-dried microcapsules exhibited a smoother thermogram with fewer discrete transitions. A broad endothermic peak appeared at 129–133 °C, attributed to the thermal response of maltodextrin and water dissociation, and no major degradation-related peaks were observed. The total enthalpy change was 20.62 J/g.

Overall, spray-dried microcapsules demonstrated a more uniform thermal transition pattern and higher thermal resistance, whereas freeze-dried samples showed lower thermal tolerance and multiple thermolabile domains.

### 2.5. Oxidative Stability of I. polycarpa Oil Microcapsules

**Figure 3:**
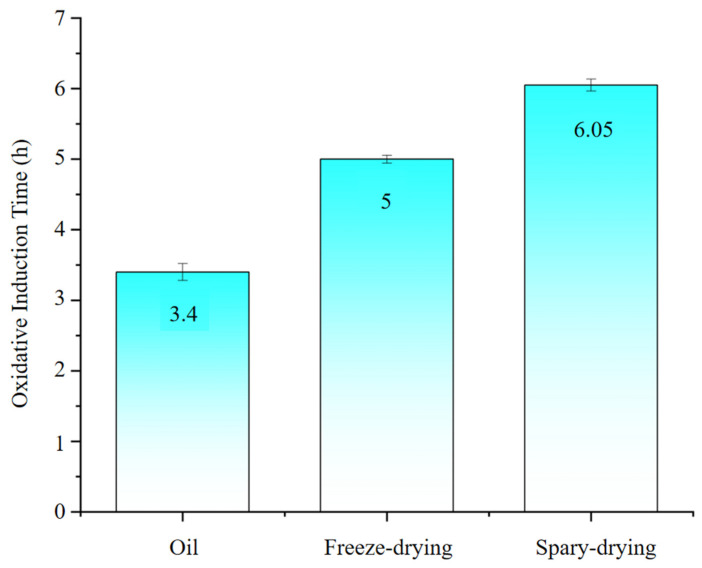
Oxidative stability index (OSI) of free *I. polycarpa* oil and its microcapsules prepared by spray drying and freeze drying.

Figure 3 shows the oxidative stability index (OSI) of *I. polycarpa* oil and its microcapsules prepared by different drying methods. Native oil exhibited the shortest induction time (3.40 ± 0.05 h), which can be attributed to the high content of polyunsaturated fatty acids that are highly prone to oxidation. After microencapsulation with SPI/MD as wall materials, the OSI values increased significantly (*p* < 0.05), demonstrating the protective effect of encapsulation. Freeze-dried microcapsules reached 5.00 ± 0.03 h, while spray-dried microcapsules achieved 6.05 ± 0.04 h, indicating superior oxidative stability. The improvement is mainly due to the physical barrier provided by the wall matrix, which reduces oxygen exposure and delays lipid peroxidation. In addition, the lower surface oil content of spray-dried microcapsules (4.0%) compared with freeze-dried microcapsules (8.8%) further contributed to their enhanced stability, as a smaller fraction of exposed oil minimizes contact with oxygen. This observation agrees with previous studies reporting a close relationship between surface oil and oxidative stability [24]. Moreover, the more porous structure generated during freeze drying may facilitate oxygen diffusion, accelerating oxidation compared with the denser matrix formed during spray drying [25]. Collectively, spray drying produced microcapsules with a more compact structure and lower surface oil, resulting in better oxidative stability than freeze drying.

### 2.6. FTIR of I. polycarpa Oil Microcapsules

**Figure 4:**
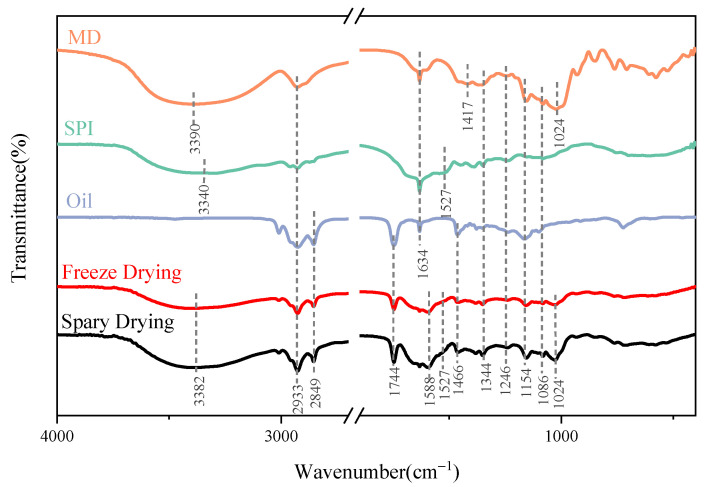
Fourier transform infrared (FTIR) spectra of wall materials, *I. polycarpa* oil, and *I. polycarpa* oil microcapsules prepared by spray drying and freeze drying.

As shown in Figure 4, FTIR was employed to analyze the molecular structures and characteristic bonds of *I. polycarpa* oil, SPI, MD, and microcapsules prepared by spray drying and freeze drying.

For MD, characteristic absorption peaks were observed at 1024 cm^−1^ and 1417 cm^−1^, corresponding to C–O–C stretching and C–H deformation vibrations, consistent with the typical spectral features of polysaccharides [14,26]. In addition, a weak band at 920 cm^−1^ was also detected, associated with C–O stretching of glycosidic bonds.

For SPI, the bands at 1634 cm^−1^ (amide I), 1527 cm^−1^ (amide II), and 1246–1344 cm^−1^ (amide III) were assigned to C=O stretching, N–H bending, and C–N/C–H vibrations of peptide bonds, respectively [27,28].

The FTIR spectrum of *I. polycarpa* oil exhibited characteristic C–H stretching peaks at 2933 cm^−1^ and 2849 cm^−1^, as well as a C=O stretching vibration at 1744 cm^−1^, attributed to ester groups or fatty acids [29,30]. The weak =C–H band at 3007 cm^−1^ further confirmed the presence of unsaturated fatty acid chains.

In the spectra of the microcapsules, both spray-dried and freeze-dried samples retained the main characteristic peaks of SPI and MD, such as the amide I band at 1634 cm^−1^, the amide II band at 1527 cm^−1^, and the C–O–C band at 1024 cm^−1^, indicating that the wall materials preserved their original chemical structures after encapsulation. The ester C=O band of the encapsulated oil became slightly broader and shifted marginally toward lower wavenumbers compared with neat oil, which can be attributed to weak hydrogen bonding or other non-covalent interactions between the oil ester groups and polar groups of SPI/MD [31]. These spectral changes support the presence of non-covalent wall–core interactions during microencapsulation rather than the formation of new covalent bonds.

Beyond the qualitative peak assignment, semi-quantitative FTIR analysis further clarified the molecular interactions within the microcapsules. The amide I band (1600–1700 cm^−1^) exhibited slight broadening compared with pure SPI, indicating partial unfolding of protein chains and enhanced hydrogen-bonding interactions with maltodextrin. The relative intensity ratio of amide I to amide II increased in the spray-dried samples, suggesting a higher proportion of ordered structures and a denser interfacial network.

Moreover, the marginal red-shift of the ester C=O band of the encapsulated oil reflects a stronger local hydrogen-bonding environment, while the more pronounced O–H stretching band indicates strengthened SPI–MD interactions within the matrix. These spectral changes collectively suggest that microencapsulation induces a tighter protein–polysaccharide network with reduced molecular mobility, helping restrict oxygen diffusion and stabilize unsaturated lipid chains.

This molecular-level interpretation supports the superior oxidative and thermal stability observed in the spray-dried microcapsules.

Overall, the FTIR analysis demonstrated that no new chemical bonds were formed in the microcapsules, and the main chemical features of *I. polycarpa* oil were preserved. Both spray drying and freeze drying effectively achieved encapsulation, while the smoother spectrum of the spray-dried sample indicated a denser and more homogeneous structure.

### 2.7. Volatile Component Analysis of I. polycarpa Oil Microcapsules

**Figure 5:**
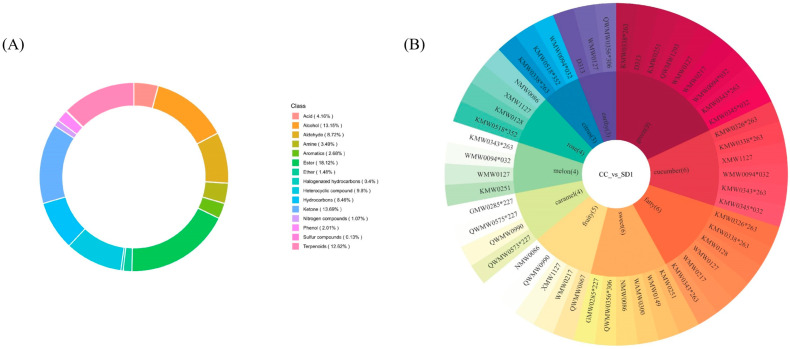
Volatile composition of *I. polycarpa* oil microcapsules: (**A**) category distribution of metabolites; (**B**) flavor wheel of differential metabolites.

Table 2 summarizes the odor activity values (OAVs) of volatile compounds identified in spray-dried and freeze-dried *I. polycarpa* oil microcapsules, while Figure 5 provides an overview of the chemical classifications of these volatiles (A) and the correlation between representative differential metabolites and sensory attributes (B).

In terms of compound distribution (Figure 5A), esters represented the dominant class (18.12%), followed by ketones (13.69%) and alcohols (13.15%), which together contributed to the essential flavor profile of the microcapsules. Terpenoids (12.62%) and heterocyclic compounds (9.8%) also played significant roles, while acids and amines, though less abundant, were crucial in fine-tuning flavor complexity.

The OAV analysis (Table 2) indicated that 14 compounds in spray-dried microcapsules and 11 compounds in freeze-dried microcapsules had OAV > 1, meaning they contributed perceptibly to the overall aroma. Notably, four key volatiles in the spray-dried samples—2-Nonenal (E), 2,6-Nonadienal (E,E), 2-Nonenal, and 2,6-Nonadienal (E,Z)—had OAV > 100, confirming their dominant role in shaping flavor. By contrast, only three compounds exceeded this threshold in freeze-dried samples, and the important cucumber-like compound 2,6-Nonadienal (E,E) was absent.

Compounds with OAV > 100 were considered key odorants contributing dominantly to the aroma profile, while compounds with OAV between 1 and 100 were regarded as secondary contributors. The sensory correlation analysis (Figure 5B) further demonstrated that differential metabolites were strongly associated with grassy (9), cucumber (6), fatty (6), and sweet (6) attributes, which are key sensory notes of *I. polycarpa* oil microcapsules. The deeper color coding for 2,6-Nonadienal (E,Z) under the “cucumber” and “green” categories confirmed its critical role in imparting intense and characteristic flavor, while its lighter association with “fatty” and “citrus” attributes suggested more delicate contributions.

Taken together, Table 2 and Figure 5 reveal that spray-dried microcapsules retained a greater number and higher intensity of key volatile compounds than freeze-dried ones, highlighting the superior ability of spray drying to preserve and enhance the characteristic aroma profile of *I. polycarpa* oil. Importantly, although the wall composition was the same, the two drying methods produced distinct microstructures: spray drying formed compact spherical shells, whereas freeze drying generated porous matrices. These structural differences likely altered the retention and release of volatile compounds, thereby contributing to the process-dependent aroma profiles observed. While minor contributions of wall-derived volatiles (e.g., pyrazines from SPI) cannot be entirely excluded, the dominant odorants with the highest OAVs (e.g., 2,6-nonadienal and 2-nonenal) are typical lipid-derived oxidation markers, confirming that the key aroma primarily originated from the core oil rather than the wall matrix.

The variation in volatile profiles between spray-dried and freeze-dried microcapsules can be attributed not only to differences in surface oil but also to structural effects on molecular diffusion and volatilization dynamics. Previous studies show that microcapsule morphology and wall matrix integrity significantly influence volatile retention: for example, Pickering emulsion-derived spray-dried microcapsules demonstrated improved flavor retention due to stable interfaces and dense shell formation, which reduced volatile loss during processing and storage [32]. Furthermore, factors such as droplet skin formation and microcapsule morphology were found to control volatile loss by modifying diffusion pathways and surface area exposure in spray-dried systems. These findings align with reviews indicating that both encapsulation conditions and wall material characteristics critically determine how effectively volatile and bioactive compounds are retained within microcapsules [33].

## 3. Discussion

### 3.1. Encapsulation Efficiency and Retention Mechanism

The higher EE observed in spray-dried microcapsules can be attributed to the rapid solvent evaporation and droplet solidification during atomization, which facilitates the prompt formation of a dense and continuous SPI/MD wall. Such a compact matrix limits oil migration toward the particle surface and reduces volatilization and oxidative losses during processing, thereby improving core retention.

In contrast, freeze drying involves a slow sublimation process that commonly yields porous and fragile matrices. The resulting structure provides less effective confinement of the oil phase and may promote partial oil leakage or redistribution to the surface, leading to a lower EE.

Similar trends have been reported in previous studies on bioactive oil microencapsulation, where spray drying generally achieves higher encapsulation efficiency than freeze drying or other low-temperature drying techniques [34,35]. Collectively, these results suggest that spray drying is more suitable when maximizing core retention and product yield is the primary objective.

### 3.2. Physicochemical Properties and Molecular-Level Interpretation

The higher solubility observed in spray-dried microcapsules can be attributed to the rapid droplet solidification during atomization, which produces smooth surfaces and improves wettability and dispersion in aqueous systems. In contrast, the slower ice sublimation in freeze drying tends to create irregular and porous structures, reducing solubility despite lower moisture content.

Moisture differences between the two methods are consistent with their drying mechanisms. Freeze drying removes water efficiently through sublimation at low temperature, whereas spray drying typically retains slightly more bound water within the carbohydrate–protein matrix.

The particle size results reflect differences in the reconstitution behavior of dried powders. Spray-dried samples exhibited larger hydrodynamic sizes, likely due to partial aggregation or the presence of compact hollow particles that swell during redispersion. In contrast, freeze-dried powders tend to break into smaller fragments during handling and hydration, resulting in smaller D10–D90 percentiles. Comparable PDI values (~0.35–0.39) indicate that both processes produced similarly broad size distributions.

ζ-potential values of both microcapsule types exceeded −30 mV, confirming adequate electrostatic stability. The slightly more negative ζ-potential for spray-dried microcapsules suggests stronger surface charge development, likely due to enhanced exposure of deprotonated SPI residues following rapid film formation during drying.

The higher emulsifying activity of freeze-dried powders reflects the greater preservation of native surface-active components under mild temperature conditions. However, the superior emulsion stability of spray-dried samples indicates the combined contribution of stronger electrostatic repulsion and the redispersion behavior reflected in DLS results.

Together, these findings demonstrate a trade-off between the two techniques: freeze drying favors lower moisture content and immediate interfacial activity, whereas spray drying provides improved solubility, more stable redispersion behavior, and enhanced electrostatic stabilization. These differences arise from the distinct microstructures formed by rapid thermal solidification versus low-temperature sublimation.

### 3.3. Morphology of I. polycarpa Oil Microcapsules

The distinct morphologies produced by the two drying methods reflect their different solidification mechanisms. During spray drying, rapid water evaporation induces instantaneous film formation at the droplet surface, generating compact spherical shells with limited shrinkage. Such dense morphologies reduce surface oil exposure and minimize interparticle fusion, contributing to higher encapsulation efficiency and enhanced oxidative stability.

In contrast, freeze drying proceeds through ice crystallization and sublimation, creating irregular porous networks as the ice templates are removed. The large voids observed in Figure 1D–F increase the internal surface area and facilitate molecular diffusion of oxygen and volatile compounds—a phenomenon commonly reported for freeze-dried biopolymer matrices. This microstructure helps to explain the higher emulsifying activity but lower oxidative stability and volatile retention of freeze-dried samples relative to spray-dried ones.

From a molecular perspective, the compact shells formed during spray drying restrict the mobility of unsaturated lipid chains and reduce the accessibility of molecular oxygen, whereas the open porous morphology of freeze-dried microcapsules provides wider diffusion pathways for oxygen and volatile molecules. These structural differences align with the process-dependent functional properties discussed in later sections, emphasizing the central role of shell density and porosity in controlling molecular diffusion, oxidation kinetics, and volatilization behavior.

### 3.4. Thermal Stability of I. polycarpa Oil Microcapsules

The thermal behaviors observed in the DSC thermograms reflect differences in the internal matrix consolidation of the two drying methods. Freeze drying tends to preserve heterogeneous domains and crystalline regions formed during slow sublimation, leading to multiple transition peaks and lower overall thermal stability. In contrast, spray drying induces rapid dehydration and solidification, producing a more compact and uniform matrix that requires greater energy to undergo thermal transitions. This explains the smoother thermogram and higher thermal resistance of the spray-dried microcapsules.

From a molecular perspective, the higher enthalpy and smoother thermal transition of the spray-dried microcapsules suggest a more compact molecular packing between SPI and MD, which limits the mobility of encapsulated lipids. The rapid dehydration during spray drying promotes closer biopolymer chain arrangement, enhancing hydrogen bonding and reducing free volume within the matrix. Such restricted molecular mobility is consistent with the higher OSI values observed, indicating that lipid radicals generated during thermal stress or oxidation are less likely to propagate within a densely packed matrix.

### 3.5. Oxidative Stability of I. polycarpa Oil Microcapsules

At the molecular level, the oxidative stability of the microcapsules can be explained by the autoxidation mechanism of polyunsaturated fatty acids. Lipid oxidation proceeds through three steps—initiation, propagation, and termination—involving hydrogen abstraction, formation of lipid radicals, and peroxyl radical chain reactions along unsaturated fatty acid chains.

The compact SPI/MD matrix formed during spray drying restricts the molecular mobility of unsaturated lipids and decreases the probability of hydrogen abstraction, thereby slowing peroxyl radical propagation. In addition, the significantly lower surface oil content reduces the number of accessible initiation sites for oxygen attack. The dense shell also limits oxygen diffusion into the core, further suppressing radical formation. In contrast, the porous and loosely connected structure generated during freeze drying provides larger diffusion pathways for oxygen, enabling faster interaction with lipid double bonds and facilitating radical propagation.

These molecular-level stabilization mechanisms collectively explain why spray-dried microcapsules exhibit the highest OSI, as both radical initiation and propagation pathways are suppressed compared with freeze-dried samples. Overall, these findings confirm that the SPI/MD wall system acts as a molecular stabilization platform by restricting lipid molecular mobility, oxygen diffusion, and radical propagation, thereby aligning the encapsulation behavior with molecular-level oxidation prevention mechanisms.

## 4. Materials and Methods

### 4.1. Raw Materials

*I. polycarpa* oil was prepared by the National Forestry and Grassland *Idesia polycarpa* Processing and Comprehensive Utilization Engineering Technology Research Center (Guiyang, China). Soybean protein isolate (SPI) was purchased from Linyi Shansong Biological Products Co., Ltd. (Linyi, China). Maltodextrin (MD) was supplied by Shandong Xiwang Sugar Co., Ltd. (Zouping, China). Sucrose fatty acid ester and glyceryl monostearate (food grade) were obtained from Guangxi Yuanchang Food Technology Co., Ltd. (Nanning, China). Analytical-grade petroleum ether, potassium bromide (KBr), and n-hexane were purchased from Tianjin Fuyu Fine Chemical Co., Ltd. (Tianjin, China).

### 4.2. Emulsion Preparation

The emulsion was prepared according to a previously reported method [36] with slight modifications. Sucrose fatty acid ester and glyceryl monostearate were used as composite emulsifiers at a mass ratio of 1:4. Soy protein isolate (SPI) and maltodextrin (MD) were selected as composite wall materials at a 1:1 mass ratio. The core material, *I. polycarpa* oil, was incorporated with the wall material at a mass ratio of 3:5. To prepare the emulsion, the composite emulsifier was dissolved in ultrapure water (65 °C) to obtain a 1.4% (*w*/*v*) emulsifier solution. The composite wall materials were then added and continuously stirred for 30 min until completely dissolved, after which the core material was added to obtain a pre-emulsion with a total solid content of 15%. The pre-emulsion was first homogenized by high-speed shearing at 10,000 rpm for 5 min and subsequently homogenized three times under 30–40 MPa using a high-pressure homogenizer. Finally, the emulsions were processed into microcapsules either by spray drying or freeze drying.

### 4.3. Spray Drying and Freeze Drying

A mini spray dryer (YC-500, Shanghai Yacheng Instrument Equipment Co., Ltd., Shanghai, China) was used for spray drying, and a freeze dryer (SCIENTZ-12N/A, Ningbo Xinzhi Biotechnology Co., Ltd., Ningbo, China) was employed for freeze drying. Prior to spray drying, the *I. polycarpa* oil microcapsule emulsion was allowed to stand overnight to remove air bubbles. The spray drying parameters were set as follows: inlet air temperature 150 °C, outlet air temperature 100 °C, and feed rate 6%. For freeze drying, the emulsions were first frozen and subsequently dried under vacuum at −54 °C for 48 h.

### 4.4. Encapsulation Efficiency

The encapsulation efficiency (EE) was determined following the method [37] with slight modifications. EE was calculated from the surface oil (SO) and total oil (TO) contents of the microcapsule samples according to Equation (1). All measurements were performed in triplicate.

(1) $$\mathrm{EE}\left(\%\right)=\frac{\mathrm{TO}-\mathrm{SO}}{\mathrm{TO}}\times 100\%$$

To determine SO, 1.0 g of microcapsules was dispersed in 30 mL of n-hexane and stirred at 60 rpm for 5 min. The oil–hexane mixture was then filtered, and the filtrate was evaporated using a rotary evaporator (R-100, Büchi, Flawil, Switzerland). The recovered oil mass after evaporation was recorded as the SO. For TO, 1.5 g of microcapsules was mixed with 4 mL of KCl solution, 8 mL of acetone, and 8 mL of chloroform, followed by stirring at 60 rpm for 30 min. The mixture was centrifuged at 10,000 rpm for 10 min, and the chloroform phase containing extracted oil was collected, filtered, and evaporated. The recovered oil mass was recorded as the TO.

### 4.5. Determination of Physicochemical Properties of the Microcapsules

#### 4.5.1. Solubility and Moisture Content

The solubility of the microcapsules was assessed following the methodology reported in [38]. Specifically, 1 g of microcapsules was placed into a Falcon tube containing 30 mL of double-distilled water, homogenized for 30 min, and centrifuged at 3000 rpm for 10 min. A 10 mL aliquot of the supernatant was transferred into a pre-dried and pre-weighed Petri dish and oven-dried at 105 °C for 4 h. Solubility (%) was calculated from the weight difference. The moisture content of the microcapsules was quantified gravimetrically according to the procedure reported in [39]. Samples were dried in a 202-1AB drying oven (Tianjin Test Instrument Co., Ltd., Tianjin, China) at 105 °C for 24 h, then cooled to room temperature in a desiccator until constant weight was achieved.

#### 4.5.2. Particle Size Distribution and Zeta Potential Measurements

Particle size distribution and ζ-potential of the microcapsule samples were measured using a nanoparticle size and potential analyzer (NS-90Z, Zhuhai OMEC Instrument Co., Ltd., Zhuhai, China). To minimize multiple scattering, samples were diluted 100-fold with deionized water. Then, 1.0 mL of the diluted dispersion was loaded into a 10 mm cuvette and analyzed in dynamic light scattering mode with 12 consecutive scans. ζ-Potential was measured using the same instrument.

#### 4.5.3. Determination of Emulsifying Activity Index and Emulsifying Stability Index

The emulsifying properties of *I. polycarpa* oil microcapsules (spray-dried and freeze-dried) were evaluated after re-dispersion in buffer as reported in [40]. Microcapsules were dispersed in 10 mM phosphate buffer (pH 7.0) at 10 mg/mL. Emulsions were prepared by mixing 10 mL of *I. polycarpa* oil with 30 mL of the microcapsule suspension (final oil volume fraction φ = 0.25) and homogenizing at 10,000 rpm for 3 min. Immediately after homogenization (0 min), 100 μL of the emulsion was withdrawn from the bottom and diluted into 10 mL of 0.1% (*w*/*v*) SDS solution. The mixture was vortexed for 20 s, and the absorbance at 500 nm was recorded with a UV-240IPC spectrophotometer (Kyoto, Japan). After 30 min, the measurement was repeated on the same sample. EAI and ESI were calculated using Equations (2) and (3):

(2) $$\mathrm{EAI}\left(m^{2}g^{-1}\right)\;=\;\left\{\left(2\;\times \;2.303\right)/\left[C\;\times \;\left(1-\varphi \right)\;\times {\;10}^{-4}\right]\right\}\;\times \;A_{500}\;\times \;\mathrm{dilution}$$

(3) $$\mathrm{ESI}\left(\%\right)=100\;\times \;\left(\frac{A}{A_{0}}\right)$$

where A_500_ is the absorbance at 500 nm measured immediately after homogenization (0 min), “dilution” is the dilution factor, C is the initial emulsion concentration (g/mL), φ is the oil volume fraction (0.25), A_0_ is the absorbance at 0 min, and A is the absorbance after 30 min.

#### 4.5.4. Microscopic Morphology

Microstructures of the microcapsule samples were observed using an optical microscope (BX53F2, OLYMPUS, Tokyo, Japan) at 4×, 10×, 20×, and 40× magnifications. Surface morphology was examined by SEM (SU8600, Hitachi, Tokyo, Japan) according to the report [15] with minor adjustments. Samples were mounted on aluminum stubs, sputter-coated with gold to improve conductivity and image quality, and imaged at 500× and 5000× magnifications with an accelerating voltage of 10 kV.

#### 4.5.5. Thermal Stability

The thermal stability of the samples was evaluated by DSC (TA Instruments, New Castle, DE, USA) under a nitrogen purge of 100 mL·min^−1^, following a reported method with minor modifications [37]. The instrument was calibrated with high-purity indium and zinc. Approximately 5.0 ± 0.1 mg of sample was weighed into an aluminum pan and hermetically sealed using a Mettler Toledo crucible-sealing press. Scans were recorded from 30 °C to 250 °C at 10 °C·min^−1^. Thermograms were analyzed to determine peak temperature (Tₚ) and enthalpy change (ΔH) from the peak area.

#### 4.5.6. Oxidative Stability

Oxidative stability of the samples was evaluated using a Rancimat instrument (873, Metrohm, Herisau, Switzerland) under accelerated conditions, following a reported procedure with minor adjustments [41]. Briefly, samples (1.5 g) were heated at 110 °C under purified air at 10 L·h^−1^. The induction time (h) obtained from the Rancimat curve was recorded as the oxidative stability index (OSI). All measurements were performed in triplicate.

#### 4.5.7. Fourier Transform Infrared Spectroscopy (FTIR)

FTIR spectra were collected to examine structural features of maltodextrin (MD), soy protein isolate (SPI), *I. polycarpa* oil, and the microcapsules prepared by spray drying and by freeze drying, following a reported procedure with minor adjustments [42]. Each sample was mixed with spectroscopic-grade KBr at a mass ratio of 1:100, ground in an agate mortar to a fine powder, and pressed at 50 MPa to form pellets. Spectra were recorded on an FTIR spectrometer (Bruker Optik GmbH, Ettlingen, Germany) over 4000–400 cm^−1^ with a resolution of 4 cm^−1^.

#### 4.5.8. Qualitative Analysis of Volatile Compounds

Volatiles were analyzed by GC/MS after headspace solid-phase microextraction following a previously reported procedure, with minor modifications [43]. Briefly, 0.2 g of the sample was placed in a 10 mL headspace vial. A pre-conditioned 50/30 μm CAR/PDMS/DVB fiber was exposed to the vial headspace while the sample was magnetically stirred in a 60 °C water bath for 30 min. The fiber was then inserted into the GC inlet for thermal desorption at 250 °C for 3 min, during which data acquisition was initiated. Chromatographic separation was performed on an Agilent 8890 GC coupled to an Agilent 7000D triple-quadrupole MS using a DB-5MS capillary column (30 m × 0.25 mm × 0.25 μm, Agilent J&W Scientific, Folsom, CA, USA). GC conditions were: carrier gas He at 1.2 mL·min^−1^; inlet 250 °C; oven program 40 → 100 °C at 3 °C·min^−1^, then 100 → 180 °C at 10 °C·min^−1^, then 180 → 280 °C at 25 °C·min^−1^, hold 5 min. The MS operated in electron-impact (EI) 70 eV mode.

### 4.6. Statistical Analysis

All measurements were performed in triplicate, and results are presented as mean ± standard deviation (SD) (n = 3). One-way ANOVA was conducted to compare treatments, followed by Duncan’s multiple range test for pairwise comparisons at *p* < 0.05. Duncan’s test was selected because its higher sensitivity allows detection of subtle differences among treatments when the sample size is small (n = 3), which is common in microencapsulation research. Although Tukey’s HSD is more conservative, Duncan’s approach provides greater statistical power for distinguishing fine variations in physicochemical and structural properties between spray-dried and freeze-dried microcapsules. Different superscript letters within the same row (or bar/point set) indicate significant differences. Graphs were prepared using Origin 8.0 (OriginLab, Northampton, MA, USA), and statistical analyses were carried out in SPSS 22.0 (IBM, Armonk, NY, USA).

## 5. Conclusions

This study compared spray drying and freeze drying for microencapsulating *Idesia polycarpa* oil using SPI/MD wall materials. Spray drying produced compact, predominantly spherical microcapsules with superior physicochemical stability and better retention of key lipid-derived odorants, whereas freeze drying yielded porous matrices with higher emulsifying activity under mild processing conditions. FTIR indicated no new covalent bonds, suggesting that wall–core interactions were mainly physical. Overall, spray drying is preferable for microcapsule applications requiring stability and aroma integrity, while freeze drying is better suited to systems prioritizing interfacial functionality or gentle handling, providing a practical basis for selecting encapsulation routes to deploy *I. polycarpa* oil across diverse food carriers.

## Figures and Tables

**Table 1:** Physicochemical properties (solubility, moisture content, ζ-potential, encapsulation efficiency, emulsifying activity, and emulsion stability) of *I. polycarpa* oil microcapsules prepared by spray drying and freeze drying.

Index	Spray Drying	Freeze Drying
Solubility (%)	51.33 ± 0.08 ^a^	47.83 ± 0.19 ^b^
Moisture content (%)	1.76 ± 0.05 ^a^	1.58 ± 0.09 ^b^
Particle size (nm)	1998.76 ± 515.77 ^a^	750.72 ± 93.55 ^b^
PDI	0.385 ± 0.23 ^a^	0.353 ± 0.22 ^a^
ζ-potential (mV)	−40.5 ± 0.70 ^b^	−36.76 ± 1.20 ^a^
Emulsifying activity (m^2^ g^−1^)	24.15 ± 0.20 ^b^	29.12 ± 0.53 ^a^
Emulsion stability (%)	95.06 ± 3.18 ^a^	88.96 ± 4.78 ^b^

Values in the same row with different superscript letters indicate statistically significant differences (*p* < 0.05).

**Table 2:** Odor activity values (OAV) of volatile compounds in spray-dried and freeze-dried *I. polycarpa* oil microcapsules.

Index	Compound	CAS	Threshold (μg/g)	OAV	
				SD	FD
KMW0326*263	2-Nonenal, (Z)-	60784-31-8	0.0045	18.4435	50.93866
KMW0338*263	2-Nonenal, (E)-	18829-56-6	0.00008	1037.447	2865.3
KMW0518*352	2,6-Octadien-1-ol, 3,7-dimethyl-, acetate, (Z)-	141-12-8	2	0.005942	0.012806
QWMW0387*306	Pyrazine, 2-butyl-3,5-dimethyl-	50888-63-6	0.18	0.33434	0.130422
QWMW0573*227	2-Cyclopenten-1-one, 3-ethyl-2-hydroxy-	21835-01-8	0.052	1.328612	0.708272
KMW0128	Hexanoic acid	142-62-1	3	0.027969	0.011969
KMW0251	5-Heptenal, 2,6-dimethyl-	106-72-9	0.016	31.55627	16.60275
KMW0382	Phenol, 2-methyl-	95-48-7	0.0039	5.093296	1.81736
NMW0058	Naphthalene, 1,2,3,4-tetrahydro-	119-64-2	0.05	0.034647	0.079618
QWMW1293	Butanoic acid, 2-hydroxy-, ethyl ester	52089-54-0	0.8	0.391698	0.155861
WAMW0698	6-Methyl-3,5-heptadiene-2-one	1604-28-0	0.1	0.425655	0.225547
WAMW2266	n-Butyl methacrylate	97-88-1	0.01	2.133274	0.978961
YZMW0438	Butanoic acid, 2-hydroxy-	565-70-8	10400	0.000005	0.000001
YZMW0482	Niacin	59-67-6	2460	0.000011	0.000004
QWM0004	Naphthalene, 1,2-dihydro-1,1,6-trimethyl-	30364-38-6	0.0025	20.61847	5.238634
WMW0149	Cedrol	77-53-2	0.0005	16.85525	55.25026
WMW0094*032	2,6-Nonadienal, (E,E)-	17587-33-6	0.0005	138.7019	29.58637
QWMW0990	Propanoic acid, 2-hydroxy-, 2-methylpropyl ester	585-24-0	340	0.000225	0.000048
WAMW0300	2(3H)-Furanone, dihydro-5-methyl-	108-29-2	0.6	0.084296	0.449525
XMW0298	Ethanol, 2-methoxy-, acetate	110-49-6	1.6	0.059551	0.012703
XMW0652	1,4-Pentanediol	626-95-9	9.9	0.004906	0.001046
YZMW0464	2-Pentanone, 4,4-dimethyl-	590-50-1	0.0081	62.29138	13.28731
YZMW0598	1H-Imidazole, 2-methyl-	693-98-1	20	0.004207	0.000897
KMW0343*263	2-Nonenal	2463-53-8	0.0001	829.9574	2292.24
KMW0530*352	Geranyl acetate	105-87-3	0.1	0.118847	0.256118
NMW0776*111	2(5H)-Furanone	497-23-4	0.06	1.160876	0.516032
QWMW0356*306	Pyrazine, 5-butyl-2,3-dimethyl-	15834-78-3	0.77	0.078157	0.030488
WAMW1251*306	Pyrazine, 3-butyl-2,5-dimethyl-	40790-29-2	2	0.030091	0.011738
QWMW0575*227	2-Hydroxy-3,5-dimethylcyclopent-2-en-1-one	21834-98-0	1	0.069088	0.03683
GMW0285*227	3,4-Dimethyl-1,2-cyclopentadione	13494-06-9	0.017	4.063989	2.16648
KMW0345*032	2,6-Nonadienal, (E,Z)-	557-48-2	0.00001	6935.096	1479.319

## Data Availability

The original contributions presented in this study are included in the article. Further inquiries can be directed to the corresponding authors.

## References

[1] Zuo Y., Liu H.B., Li B., Zhao H., Li X.L., Chen J.T., Wang L., Zheng Q.B., He Y.Q., Zhang J.S. (2024). The *Idesia polycarpa* genome provides insights into its evolution and oil biosynthesis. Cell Rep..

[2] Li Y., Peng T., Huang L., Zhang S.Y., He Y.Y., Tang L. (2019). The evaluation of lipids raw material resources with the fatty acid profile and morphological characteristics of *Idesia polycarpa* Maxim. var. vestita Diels fruit in harvesting. Ind. Crops Prod..

[3] Wen L.Y., Xiang X.W., Wang Z.R., Yang Q.Q., Guo Z.H., Huang P.M., Mao J.M., An X.F., Kan J.Q. (2022). Evaluation of cultivars diversity and lipid composition properties of *Idesia polycarpa* var. *vestita* Diels. J. Food Sci..

[4] Guo X.N., Zhang Q., Chen Y.B., Huang X.F., Yang W.Q., Li S., Li S.Y., Luo K., Xin X.L. (2023). A systematic study on composition and antioxidant of 15 varieties of wild *Idesia polycarpa* fruits in China. Front. Sustain. Food Syst..

[5] Huang L., Zeng Y., Li F., Zheng X., Rao Q., Gajendran B., Varier K.M., Peng T., Tang L. (2023). Polyphenolic compounds from *Idesia polycarpa* Maxim. fruits ameliorate non-alcoholic fatty liver disease by modulating lipid metabolism in oleic acid-induced HepG2 cells and high-fat diet-induced mice. J. Funct. Foods.

[6] Ye Y., Jia R.R., Tang L., Chen F. (2014). In Vivo Antioxidant and Anti-Skin-Aging Activities of Ethyl Acetate Extraction from *Idesia polycarpa* Defatted Fruit Residue in Aging Mice Induced by D-Galactose. Evid.-Based Complement. Altern. Med..

[7] Koç M., Güngör Ö., Zungur A., Yalçin B., Selek I., Ertekin F.K., Ötles S. (2015). Microencapsulation of Extra Virgin Olive Oil by Spray Drying: Effect of Wall Materials Composition, Process Conditions, and Emulsification Method. Food Bioprocess Technol..

[8] Lim W.T., Nyam K.L. (2016). Characteristics and controlled release behaviour of microencapsulated kenaf seed oil during in-vitro digestion. J. Food Eng..

[9] Hu X., Liu L., Zhong J.F., Liu X., Qin X.L. (2024). Improved physicochemical properties and in vitro digestion of walnut oil microcapsules with soy protein isolate and highly oxidized konjac glucomannan as wall materials. Food Chem..

[10] Karrar E., Mahdi A.A., Sheth S., Ahmed I.A.M., Manzoor M.F., Wei W., Wang X.G. (2021). Effect of maltodextrin combination with gum arabic and whey protein isolate on the microencapsulation of gurum seed oil using a spray-drying method. Int. J. Biol. Macromol..

[11] Zhang J., Zhang C., Chen X., Quek S.Y. (2020). Effect of spray drying on phenolic compounds of cranberry juice and their stability during storage. J. Food Eng..

[12] Wang M.M., Mu H.Y., Peng L., Tan C.L., Chen Y.Y., Sheng J., Tian Y., Zhao C.C. (2024). Effect and application of spray drying and freeze drying on characterization of walnut oil microcapsules. J. Food Eng..

[13] da Silva Júnior M.E., Araújo M.V.R.L., Martins A.C.S., Lima M.D.S., da Silva F.L.H., Converti A., Maciel M.I.S. (2023). Microencapsulation by spray-drying and freeze-drying of extract of phenolic compounds obtained from ciriguela peel. Sci. Rep..

[14] Zhou D., Pan Y., Ye J., Jia J., Ma J., Ge F. (2017). Preparation of walnut oil microcapsules employing soybean protein isolate and maltodextrin with enhanced oxidation stability of walnut oil. LWT-Food Sci. Technol..

[15] Tao Y.T., Tang Z.H., Huang Q.Y., Xu X.F., Cheng X.Y., Zhang G.X., Jing X.Y., Li X.L., Liang J., Granato D. (2024). Effects of spray drying temperature on physicochemical properties of grapeseed oil microcapsules and the encapsulation efficiency of pterostilbene. LWT-Food Sci. Technol..

[16] Yaman D.M., Yanik D.K., Demir A.E., Karka H.U., Güçlü G., Selli S., Kelebek H., Gögüs F. (2023). Effect of Encapsulation Techniques on Aroma Retention of *Pistacia terebinthus* L. Fruit Oil: Spray Drying, Spray Freeze Drying, and Freeze Drying. Foods.

[17] Stabrauskiene J., Pudziuvelyte L., Bernatoniene J. (2024). Optimizing Encapsulation: Comparative Analysis of Spray-Drying and Freeze-Drying for Sustainable Recovery of Bioactive Compounds from *Citrus x paradisi* L. Peels. Pharmaceuticals.

[18] Koo H., Kim S., Lee J. (2023). Comparison of physicochemical properties and oxidative stability of microencapsulated perilla oil powder prepared by freeze-drying and spray-drying. Food Sci. Biotechnol..

[19] Ledri S.A., Milani J.M., Shahidi S.A., Golkar A. (2024). Comparative analysis of freeze drying and spray drying methods for encapsulation of chlorophyll with maltodextrin and whey protein isolate. Food Chem. X.

[20] Wang T., Chen K.R., Zhang X.Z., Yu Y.J., Yu D.A.Y., Jiang L.Z., Wang L.Q. (2021). Effect of ultrasound on the preparation of soy protein isolate-maltodextrin embedded hemp seed oil microcapsules and the establishment of oxidation kinetics models. Ultrason. Sonochem..

[21] Yu Z., Garcia A.S., Johnston K.P., Williams R.O. (2004). Spray freezing into liquid nitrogen for highly stable protein nanostructured microparticles. Eur. J. Pharm. Biopharm..

[22] Tang C.-H., Choi S.-M., Ma C.-Y. (2007). Study of thermal properties and heat-induced denaturation and aggregation of soy proteins by modulated differential scanning calorimetry. Int. J. Biol. Macromol..

[23] Elnaggar Y.S.R., El-Massik M.A., Abdallah O.Y., Ebian A.E.R. (2010). Maltodextrin: A novel excipient used in sugar-based orally disintegrating tablets and phase transition process. Aaps Pharmscitech.

[24] Timilsena Y.P., Adhikari R., Barrow C.J., Adhikari B. (2016). Microencapsulation of chia seed oil using chia seed protein isolate-chia seed gum complex coacervates. Int. J. Biol. Macromol..

[25] Rahman M.S., Al-Belushi R.M., Guizani N., Al-Saidi G.S., Soussi B. (2009). Fat oxidation in freeze-dried grouper during storage at different temperatures and moisture contents. Food Chem..

[26] Zhao Y., Wang N., Pang S.F., Zhang Y.H. (2016). In-situ micro-FTIR spectroscopic observation on the hydration process of *Poria cocos*. Spectrochim. Acta Part A-Mol. Biomol. Spectrosc..

[27] Teng Z., Luo Y., Wang Q. (2012). Nanoparticles synthesized from soy protein: Preparation, characterization, and application for nutraceutical encapsulation. J. Agric. Food Chem..

[28] Rani S., Kumar K.D., Mandal S., Kumar R. (2020). Functionalized carbon dot nanoparticles reinforced soy protein isolate biopolymeric film. J. Polym. Res..

[29] Barbosa A.E.G., Constantino A.B.T., Bastos L.P.H., Garcia-Rojas E.E. (2022). Encapsulation of sacha inchi oil in complex coacervates formed by carboxymethylcellulose and lactoferrin for controlled release of β-carotene. Food Hydrocoll. Health.

[30] Aremo A.-A.E., Oluwadare A.O., Aremo J.O., Celik H., Heyne J., Han Y., Simmons B.A. (2025). Characterization of Kariya (*Hildegardia barteri* (Mast.) Kosterm) Seed Oil Fatty Acid Methyl Ester Prepared from Basic Catalytic Transesterification. Sustainability.

[31] Mansour M., Salah M., Xu X. (2020). Effect of microencapsulation using soy protein isolate and gum arabic as wall material on red raspberry anthocyanin stability, characterization, and simulated gastrointestinal conditions. Ultrason. Sonochem..

[32] Burgos-Diaz C., Leal-Calderon F., Mosi-Roa Y., Chacon-Fuentes M., Garrido-Miranda K., Opazo-Navarrete M., Quiroz A., Bustamante M. (2024). Enhancing the Retention and Oxidative Stability of Volatile Flavors: A Novel Approach Utilizing O/W Pickering Emulsions Based on Agri-Food Byproducts and Spray-Drying. Foods.

[33] Mohammed N.K., Tan C.P., Manap Y.A., Muhialdin B.J., Hussin A.S.M. (2020). Spray Drying for the Encapsulation of Oils—A Review. Molecules.

[34] Muhoza B., Yuyang H., Uriho A., Harindintwali J.D., Liu Q., Li Y. (2023). Spray-and freeze-drying of microcapsules prepared by complex coacervation method: A review. Food Hydrocoll..

[35] Chaji S., Zenasni W., Ouaabou R., Ajal E., Lahlali R., Fauconnier M.L., Hanine H., Cerne M., Paskovic I., Merah O. (2024). Nutrient and Bioactive Fraction Content of *Olea europaea* L. Leaves: Assessing the Impact of Drying Methods in a Comprehensive Study of Prominent Cultivars in Morocco. Plants.

[36] Su K., Liu Y., Song H.L. (2017). Identification of Xiangzaolu Key Aroma Compounds and Stability Analysis of Microcapsule Production of Simulated Substance. J. Food Process. Preserv..

[37] Napiorkowska A., Szpicer A., Wojtasik-Kalinowska I., Perez M.D.T., Gonzalez H.D., Kurek M.A. (2023). Microencapsulation of Juniper and Black Pepper Essential Oil Using the Coacervation Method and Its Properties after Freeze-Drying. Foods.

[38] Cano-Chauca M., Stringheta P., Ramos A., Cal-Vidal J. (2005). Effect of the carriers on the microstructure of mango powder obtained by spray drying and its functional characterization. Innov. Food Sci. Emerg. Technol..

[39] Mehyar G.F., Al-Isamil K.M., Al-Ghizzawi H.M., Holley R.A. (2014). Stability of Cardamom (*Elettaria cardamomum*) Essential Oil in Microcapsules Made of Whey Protein Isolate, Guar Gum, and Carrageenan. J. Food Sci..

[40] Wang X., Yue C.H., Xu H.H., Guan C., Guo R.C., Yang X.T., Ma C.H., Shao M.L. (2021). Comparison of emulsifying properties of fibrils formed from whey protein concentrate following induction by nuclei and nuclei fragments. Int. Dairy J..

[41] Comunian T.A., Gomez-Estaca J., Ferro-Furtado R., Conceicao G.J., Moraes I.C., de Castro I.A., Favaro-Trindade C.S. (2016). Effect of different polysaccharides and crosslinkers on echium oil microcapsules. Carbohydr. Polym..

[42] Ren X., Hou T., Liang Q., Zhang X., Hu D., Xu B., Chen X., Chalamaiah M., Ma H. (2019). Effects of frequency ultrasound on the properties of zein-chitosan complex coacervation for resveratrol encapsulation. Food Chem..

[43] Wang L., Zhang Y., Chen J.F., Luo Y.Y., Zou C.X., Qin L.K., Jia Y.L. (2023). Study on preparation and properties of *Camellia oleifera* seed oil microcapsules by complex coacervation and spray drying. LWT-Food Sci. Technol..

